# Public Transportation Use, Sexual Harassment, and Mental Health in Adults from the General Population: A Bayesian Network Analysis

**DOI:** 10.3390/ejihpe15110224

**Published:** 2025-10-29

**Authors:** Jonatan Baños-Chaparro, Diego Valencia-Pecho, Tomás Caycho-Rodríguez, Esteban Sarmiento-Suarez, Dulce Bernabel-Tarazona, Gabriela Rivera-Álvarez, Julio Torales

**Affiliations:** 1 Programa académico de Psicología, Facultad de Ciencias de la Salud, Universidad Privada Norbert Wiener, Lima 15046, Peru; diego.valencia@uwiener.edu.pe (D.V.-P.); a2023102054@uwiener.edu.pe (E.S.-S.); a2024102782@uwiener.edu.pe (D.B.-T.); a2023102380@uwiener.edu.pe (G.R.-Á.); 2 Facultad de Psicología, Universidad Científica del Sur, Lima 15046, Peru; tcaycho@cientifica.edu.pe; 3 Facultad de Ciencias Médicas, Universidad Nacional de Asunción, San Lorenzo 16601, Paraguay; jtorales@fcmuna.edu.py; 4 Facultad de Ciencias de la Salud, Universidad de Los Lagos, Osorno 5290000, Chile; 5 Facultad de Ciencias de la Salud, Universidad Sudamericana, Pedro Juan Caballero 130105, Paraguay

**Keywords:** transportation, urban mobility, sexual harassment, mental health, adults

## Abstract

**Background**: Public transportation is one of the primary modes of mobility in urban environments, but it has also become a setting where sexual harassment frequently occurs. This situation not only compromises users’ safety but also has a significant impact on mental health. The objective of the study is to analyze the relationship between public transportation use, sexual harassment, and mental health through directed and undirected network analyses. **Methods**: This was an associative, basic, quantitative, and cross-sectional study. A total of 507 Peruvian adults (66.7% women) participated by completing a sociodemographic questionnaire and using psychological instruments. A Gaussian graphical model and a directed acyclic graph were used to estimate the networks, including assessments of precision and stability. **Results**: Results indicated that in the undirected network, public transportation use was associated with sexual harassment and anger. The central symptoms were generalized anxiety and depression. In the directed network, public transportation use influenced both sexual harassment and generalized anxiety. Furthermore, distress and sexual harassment emerged as conditionally dependent on multiple psychological factors. **Conclusions**: The findings suggest that implementing preventive and psychosocial intervention strategies in the context of public transportation may reduce experiences of sexual harassment and the manifestation of other mental health problems among adults in the general population, thereby promoting safer and healthier public environments.

## 1. Introduction

Public transportation is a fundamental aspect of urban mobility, facilitating the movement of millions of people around the world for work, education, and social activities. However, frequent use of public transportation has been associated with a range of issues that negatively impact users’ mental health, particularly in densely populated cities (Iamtrakul et al., 2024; Ye et al., 2023). Factors such as overcrowding, delays, insecurity, and sexual harassment significantly and directly or indirectly affect individuals’ psychological well-being (Boyer, 2021).

In recent decades, sexual harassment on public transportation has increased in urban settings worldwide. Recent studies have reported prevalence rates ranging from 40% to 89% among general and university populations, while an international review found rates between 15% and 95%, with particularly high levels in Asia and Africa. These incidents not only violate victims’ rights but also have serious mental health consequences (Gekoski et al., 2017; Loukaitou-Sideris et al., 2020; Nguyen et al., 2025; Tabassum & Suhail, 2022). Such experiences can lead to a perception of insecurity during travel, which, in turn, results in decreased use of public transportation and negatively affects users’ well-being and quality of life (Anwar et al., 2022; Nath et al., 2022; Östergren et al., 2022). The normalization of harassment in public transportation and the lack of effective reporting strategies exacerbate the problem, creating environments in which victims feel vulnerable.

Furthermore, the relationship between public transportation use and mental health is well-documented in empirical research. Public transportation can be a source of stress and anxiety, particularly in situations involving traffic congestion, noise exposure, lack of safety, poor hygiene, and general dissatisfaction (Boyer, 2021; Torales et al., 2024). These conditions may significantly affect mental health. Studies have shown that the use of public transportation, especially during long or crowded journeys, is associated with psychological problems such as stress, generalized anxiety, depressive symptoms, and anger, which are potential risk factors for suicidal ideation (Iamtrakul et al., 2024; Lan et al., 2020; Wicki et al., 2023; Ye et al., 2023). Time spent commuting has been correlated with lower overall satisfaction and increased psychological difficulties (Conceição et al., 2023; Torales et al., 2024). The quality, accessibility, and perceived safety of public transportation are key factors shaping users’ travel experiences and their impact on mental health.

From a theoretical perspective, the relationship between public transport use, sexual harassment, and mental health can be understood through the ecological model of human behavior, which emphasizes that individuals’ psychological experiences are shaped by the interaction of multiple levels: individual, interpersonal, community, and societal. In this sense, public transport functions as a microsystem where structural factors (safety conditions, accessibility), interpersonal dynamics (interactions with other users, risk of harassment), and individual aspects (perceived insecurity, coping strategies) converge. These levels interact dynamically and produce cumulative effects on users’ psychological well-being (Gekoski et al., 2017; Loukaitou-Sideris et al., 2020). At the same time, the biopsychosocial model of mental health posits that environmental stressors, such as recurrent exposure to harassment or poor transport conditions, interact with psychological and social vulnerabilities to trigger or exacerbate symptoms such as anxiety, depression, or suicidal ideation (Lan et al., 2020; Wicki et al., 2023; Torales et al., 2024). Thus, the combination of both models offers an integrative framework that recognizes the multicausality of these phenomena and the need for methodologies capable of capturing it.

In this context, psychological network analysis, both in its undirected Gaussian variant and in directed Bayesian networks, emerges as a particularly suitable methodological tool. The logic of networks naturally reflects the ecological structure: nodes represent variables located at different levels (individual, interpersonal, and contextual), while edges illustrate how these interact with one another. Moreover, Bayesian models allow for the exploration of probabilistic pathways that align with the biopsychosocial perspective by showing how contextual stressors increase the likelihood of psychological symptoms. In this way, the identification of central nodes, such as anxiety or depression, not only provides descriptive information but also reveals mechanisms through which the public transport environment affects mental health, in coherence with the proposed theoretical frameworks.

Despite growing evidence on the negative effects of public transportation on mental health and the high prevalence of sexual harassment in these settings, a significant fragmentation persists within the scientific literature. Studies tend to address these phenomena in isolation, on the one hand, analyzing transport quality and its psychological effects, and on the other, examining gender-based violence in public spaces without considering that these experiences often occur simultaneously and generate interdependent impacts on users’ well-being. This lack of integration limits a deeper understanding of how these variables interact, reinforce, or modulate each other within complex urban contexts. Understanding these interactions requires moving beyond traditional linear approaches and adopting models capable of capturing the dynamic and multicausal nature of these phenomena. Within this framework, psychological network analysis emerges as an innovative and effective methodological tool, allowing complex systems to be represented through nodes (variables) and edges (connections), thereby facilitating the identification of dependency patterns, underlying structures, and potential pathways of influence between individual and contextual variables (Isvoranu et al., 2022).

A network is composed of nodes (psychological variables) and edges (connections between pairs of nodes). There are several subtypes of network models, including undirected Gaussian networks, which estimate partial correlations between pairs of nodes without assuming directionality. This type of network is useful for exploring patterns of statistical dependence and for visualizing contemporary relationships among psychological variables (Isvoranu & Epskamp, 2023; Isvoranu et al., 2022). In contrast, directed Bayesian networks allow for modeling probabilistic relationships with directionality, making them suitable for inferring potential causal pathways between nodes (Briganti et al., 2023; Sonis & Jiang, 2023). The combination of both approaches provides a comprehensive understanding of the psychological dynamics involved in complex social contexts.

To date, few studies have attempted to integrate public transportation use, sexual harassment, and mental health within a single analytical model. The limited research that does address these interrelations tends to rely on traditional quantitative approaches, without capturing the potential interdependent connections between variables. Therefore, there is an urgent need to approach this phenomenon from a more integrative and complex perspective.

In this context, the aim of the present study was to analyze the relationships between public transportation use, sexual harassment, and mental health through both directed and undirected network analyses, using a representative sample of the general adult population. This approach allows for the exploration of how these variables interact with one another and identifies the most central nodes within the network, providing valuable evidence to inform public policy and intervention strategies.

## 2. Materials and Methods

### 2.1. Participants

This research employed an associative, quantitative, and cross-sectional design. An online survey was conducted between January and April 2025 using a Google Forms questionnaire. The survey was distributed through the researchers’ social media platforms and included information about the study’s objectives, anonymity, academic purpose, data handling, and informed consent. Internet-based surveys provide greater access to study samples, systematic control over responses, the use of multiple media formats, and efficient and cost-effective administration (Gosling & Mason, 2015).

A total of 507 Peruvian adults from the general population participated in the study. The majority were women (66.7%), with a mean age of 33 years (SD = 12.05; range = 18 to 60 years). Furthermore, 72.4% reported being currently employed, while 27.6% were unemployed. Regarding marital status, most participants identified as single (72%) or married (25%), followed by divorced (2.8%) and widowed (0.2%). Most participants resided in urban areas (95.9%). In terms of educational level, incomplete university studies (32.7%), completed university education (26.2%), and completed technical education (12.2%) were most frequently reported.

### 2.2. Measures

#### 2.2.1. Demographic Information

A brief demographic questionnaire was used to gather information on gender, age, employment status, marital status, area of residence, educational level, average commute time using public transportation (round trip), overall satisfaction with public transportation services (general, hygiene, comfort, safety, and driver behavior), and experience of sexual harassment in the past twelve months (within public transportation, type of transport, and type of perpetrator).

#### 2.2.2. Questionnaire on the Use of Public Transportation and Well-Being (QUPTW)

This is a nine-item questionnaire that assesses perceptions of public transportation use and its relationship with well-being (Torales et al., 2024). Each item is rated using a five-point Likert scale ranging from 0 (strongly disagree) to 4 (strongly agree). The total score ranges from 9 to 45, with higher scores indicating poorer well-being. In this study, its psychometric properties were examined, revealing a unidimensional factorial structure (CFI = 0.99, TLI = 0.99, RMSEA = 0.07 [95% CI: 0.055, 0.086], SRMR = 0.046) and adequate reliability (ω = 0.88).

#### 2.2.3. Dimensions of Anger Reactions-5 (DAR-5)

The DAR-5 is a five-item scale that evaluates experiences of anger over the past four weeks. It uses a five-point Likert scale ranging from 1 (none or almost none of the time) to 5 (all or almost all of the time). Total scores range from 5 to 25, with higher scores indicating more frequent experiences of anger. The Peruvian adaptation was used, and this study showed adequate reliability (ω = 0.86) (Caycho-Rodríguez et al., 2020).

#### 2.2.4. Perceived Stress Scale (PSS)

This 12-item scale measures perceived stress during the past month and consists of two dimensions: eustress and distress. It includes both positively and negatively worded statements about stressful situations or life events. Responses are given on a Likert scale from 0 (never) to 4 (very often), with positively worded items reverse scored. Total scores range from 0 to 48, with higher scores indicating greater perceived stress. The Peruvian adaptation was used, and this study reported adequate reliability for the distress dimension (ω = 0.86) and the eustress dimension (ω = 0.92) (Boluarte-Carbajal et al., 2023).

#### 2.2.5. Frequency of Suicidal Ideation Inventory (FSII)

This inventory assesses the frequency of suicidal ideation over the past year using five items. Items are rated on a Likert scale ranging from 1 (never) to 5 (almost every day). The total score ranges from 5 to 25, with higher scores indicating greater frequency of suicidal ideation. The adapted Peruvian version was used, and this study found acceptable reliability (ω = 0.96) (Baños-Chaparro et al., 2021).

#### 2.2.6. Patient Health Questionnaire-2 (PHQ-2)

The PHQ-2 is a brief two-item questionnaire that assesses depressive symptoms over the past two weeks. Each item is rated on a four-point scale from 0 (not at all) to 3 (nearly every day). The total score ranges from 0 to 6, with higher scores indicating greater depressive symptomatology. The Peruvian adaptation was used, and this study reported good reliability (ω = 0.73) (Villarreal-Zegarra et al., 2023).

#### 2.2.7. Generalized Anxiety Disorder-2 (GAD-2)

The GAD-2 is a short scale assessing generalized anxiety over the past two weeks through two items. Each item is rated on a four-point scale from 0 (not at all) to 3 (nearly every day). The total score ranges from 0 to 6, with higher scores reflecting greater levels of generalized anxiety. The Peruvian adaptation was used, and this study reported adequate reliability (ω = 0.88) (Franco-Jimenez & Nuñez-Magallanes, 2022).

### 2.3. Statistical Analysis

Statistical analyses were conducted using RStudio software, version 4.3.2. In the first stage, descriptive statistics such as mean and standard deviation were calculated to summarize the average scores. Before constructing the network, redundancy among nodes was examined using the goldbricker function from the *networktools* package version 1.5.0, identifying node pairs with more than 25% topological overlap, using a significance threshold of *p* = 0.05 (Hittner et al., 2003; Jones, 2021).

The estimation of the undirected network was conducted in three stages. First, an unregularized network model was generated using the estimateNetwork function from the *bootnet* package version 1.5.3, implementing the *ggmModSelect* algorithm with Spearman correlations, which is appropriate for handling non-symmetric data (Isvoranu & Epskamp, 2023). This algorithm selects the optimal Gaussian graphical model by evaluating 100 random models and applying the Extended Bayesian Information Criterion (EBIC). Unregularized methods have shown adequate performance when the number of participants exceeds the number of nodes included in the network and when the research question involves analyzing edges between node pairs and the centrality of the underlying network structure (Burger et al., 2023; Isvoranu & Epskamp, 2023; Williams et al., 2019). For visualization, the *qgraph* package version 1.9.5 was used along with the Fruchterman–Reingold algorithm, representing nodes as circles and conditional associations as lines, where positive connections are shown in blue and negative ones in red. The thickness and color intensity reflect the strength of the associations (Epskamp et al., 2012; Fruchterman & Reingold, 1991).

In the second stage, both local and global network properties were evaluated. At the local level, Expected Influence (EI) was estimated using the centrality function from the *qgraph* package, reflecting the accumulated relevance of each node while considering the direction of connections (Epskamp et al., 2012). Predictability was also evaluated through explained variance (*R*^2^) using the predict function from the *mgm* package version 1.2 (Haslbeck & Waldorp, 2018, 2020). This measure indicates the extent to which a node can be predicted by its neighboring connections, providing a practical estimate of its importance. For global properties, three metrics were calculated: density (D), representing the average strength of connections between nodes; global transitivity (C△), indicating the clustering tendency of the network; and average path length (APL), which reflects the efficiency of information transmission. Additionally, the small-world index (S) was computed, where values greater than 1 suggest a highly connected network with significant clustering structures (Isvoranu et al., 2022). These metrics were obtained using the smallworldIndex function from the *qgraph* package (Epskamp et al., 2012).

Finally, in the third stage, the precision and stability of the network were examined through non-parametric Bootstrap resampling techniques. A total of 1000 Bootstrap samples were generated using the *bootnet* package version 1.5.3, calculating 95% confidence intervals (CIs) for each edge (Burger et al., 2023). For stability assessment, a case-dropping procedure was applied, re-estimating the network after randomly removing participants. The correlation stability coefficient (SC) was computed, indicating the maximum proportion of cases that can be dropped while maintaining stable EI estimates. A SC value above 0.25 is considered acceptable (Burger et al., 2023; Isvoranu et al., 2022).

Separately, the Bayesian network was constructed using a Directed Acyclic Graph (DAG) model via the bnlearn package version 4.8.3 (Scutari, 2010). In this model, nodes are connected by directional arrows indicating probabilistic dependencies between variables. For example, a connection A → B implies a direct effect of A on B, while a chain A → B → C suggests an indirect effect of A on C through B. Arrows represent probabilistic causality between variables, while their absence implies statistical independence and no directionality (Sonis & Jiang, 2023).

DAG estimation was performed using a score-based structural learning approach, applying the Hill-Climbing (HC) algorithm with the *hc* function. This algorithm identifies the optimal structure by iteratively adding, removing, or reversing edges, exploring all possible network combinations. Model selection is guided by optimization of the Bayesian Information Criterion (BIC), with lower BIC values indicating a better-fitting model (Briganti et al., 2023; Scutari, 2010; Sonis & Jiang, 2023). The procedure included 50 random restarts and 100 perturbations per restart (Briganti et al., 2023).

To assess DAG stability, the procedure proposed by Briganti et al. (2023) was followed in three steps: first, 10,000 *Bootstrap* samples were generated with five random restarts and 10 perturbations each using the *boot.strength* function; second, the structural learning algorithm was reapplied to each sample; and third, the resulting structures were averaged to obtain a consolidated DAG. Additionally, a data-driven thresholding method by Scutari and Nagarajan (2013) was used to retain edge frequencies, and directionality was determined by retaining those edges that appeared in the same direction in at least 50% of the Bootstrap networks (Briganti et al., 2023).

For easier interpretation, two visualizations were created using the *strength.plot* function. The first graph shows DAG connections weighted by their BIC values, where thicker arrows indicate greater contributions to model fit. The second graph illustrates directional probabilities, with thicker arrows representing a higher proportion of that direction’s presence in the Bootstrap-generated networks (Briganti et al., 2023).

### 2.4. Ethical Considerations

The study adhered to both international and national ethical guidelines in psychological research. Participants provided informed consent prior to participation. The survey was anonymous, voluntary, and data confidentiality was assured (Hilbig et al., 2022). Additionally, the study was reviewed and approved by the ethics committee of the Universidad Privada Norbert Wiener under registration number 0833-2024-CIEIC-UPNW.

## 3. Results

### 3.1. Prevalence of Public Transportation Use and Satisfaction

The average travel time using public transportation (including round trips) was most commonly reported as between 1 and 1.9 h (33.7%, *n* = 171), followed by less than 1 h (27.6%, *n* = 140), 2 to 2.9 h (20.9%, *n* = 106), 3 to 3.9 h (10.8%, *n* = 55), more than 4 h (5.5%, *n* = 28), and “don’t remember” (1.4%, *n* = 7). Additionally, 22.5% of participants reported having been victims of sexual harassment (*n* = 114). These incidents occurred in different types of public transport, including minivans or “combis” (8.3%), buses (5.1%), and the Metropolitan system (3.4%). Simultaneous incidents were also reported across multiple transport modes, such as combi and bus (2.2%), combi and train (1.2%), and combi and motorcycle taxi (1.2%). In terms of perpetrators, most were passengers (19.5%) and drivers (1.6%). There were also combinations, such as passenger and fare collector (3%), fare collector and driver (1.4%), and passenger, collector, and driver (1%).

Regarding satisfaction with public transportation, Table 1 shows that general satisfaction, hygiene, comfort, and driver behavior were most frequently rated as fair or poor. Notably, satisfaction with safety was rated worse than the other dimensions, with a higher proportion of participants rating it as poor rather than fair. Importantly, excellent satisfaction was absent in all categories.

### 3.2. Global Network Properties

The network exhibited a density of 0.064, indicating a total of 16 connections, of which 12 were positive and 4 negative. The nodes showed a strong tendency to form clusters (*C△* = 0.58), surpassing the expected value by chance (*C△*_(random)_ = 0.50). The average number of steps required to transmit information between pairs of nodes was 1.46. Lastly, the small-world index (*S*) reached a value of 1.25, suggesting that the symptom network possesses typical small-world characteristics.

### 3.3. Local Network Properties

Regarding descriptive statistics, Table 2 shows that the highest mean and standard deviation were found for the use of public transportation (*M* = 32.15, *SD* = 7.04), while the lowest were found for sexual harassment (*M* = 0.78, *SD* = 0.42). The node redundancy analysis revealed no suggestions for removal, as no pairs of nodes were identified as redundant.

**Figure 1:**
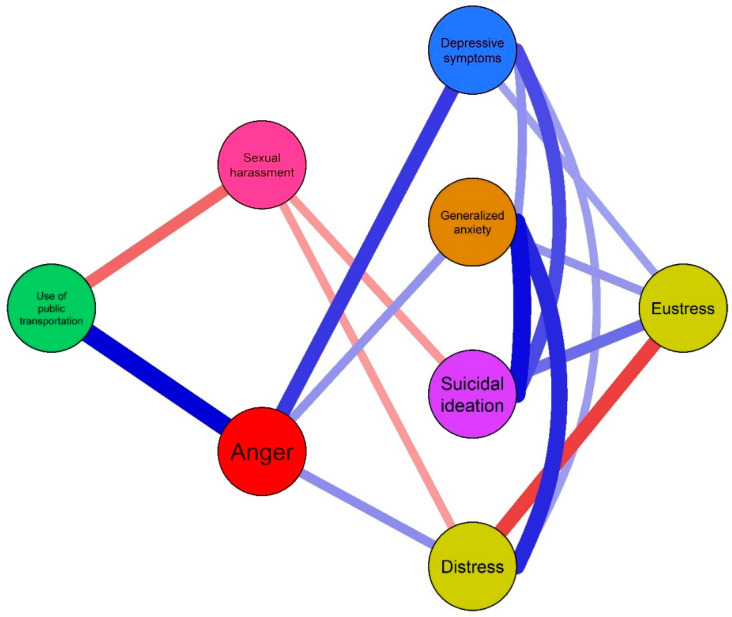
Flowchart of the network structure of public transportation use, sexual harassment, and mental health among adults from the general population.

In terms of local network properties, the most central nodes in terms of Expected Influence (EI) were generalized anxiety (*EI* = 1.05), depressive symptoms (*EI* = 0.93), and anger (*EI* = 0.91). Regarding predictability, the most predictable nodes were sexual harassment (79.3%) and generalized anxiety (41.7%) (see Table 2). The strongest relationships in the network structure, shown in Figure 1, were between public transportation use and anger (*r* = 0.34), suicidal ideation and generalized anxiety (*r* = 0.32), distress and generalized anxiety (*r* = 0.29), anger and depressive symptoms (*r* = 0.27), distress and eustress (*r* = −0.26), and public transportation use and sexual harassment (*r* = −0.21).

### 3.4. Precision and Stability of the Network Structure

**Figure 2:**
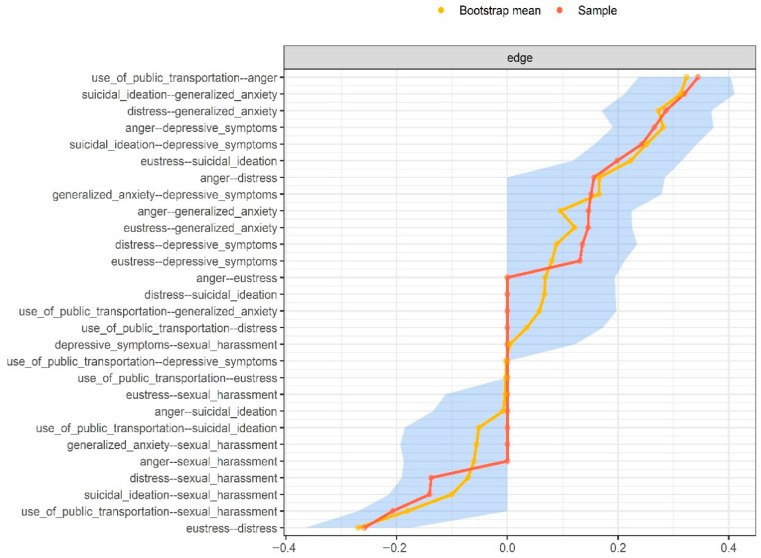
Nonparametric bootstrap confidence intervals of estimated edges for the network structure.

**Figure 3:**
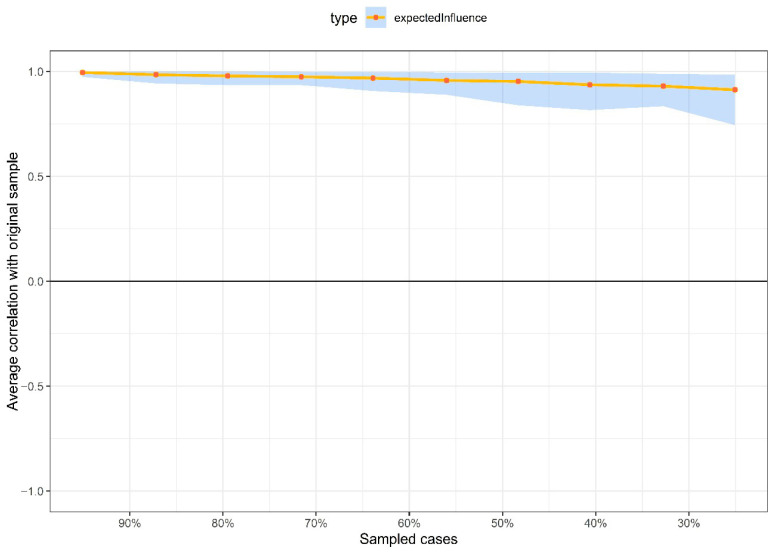
Stability of the expected influence centrality index.

Figure 2 presents the precision of the edges. Overall, the confidence intervals (CIs) surrounding both the original sample estimates and the resampling-based means were narrow and consistent for most connections. However, some edges lacked confidence intervals, reflecting minor variations in the resampling process. On the other hand, Figure 3 illustrates the stability of Expected Influence (EI), where the analysis based on progressively dropping percentages of the original sample showed good stability (SC = 0.75 [Minimum = 0.673, Maximum = 1]), indicating that the results are robust and interpretable.

### 3.5. Bayesian Network Structure

**Figure 4:**
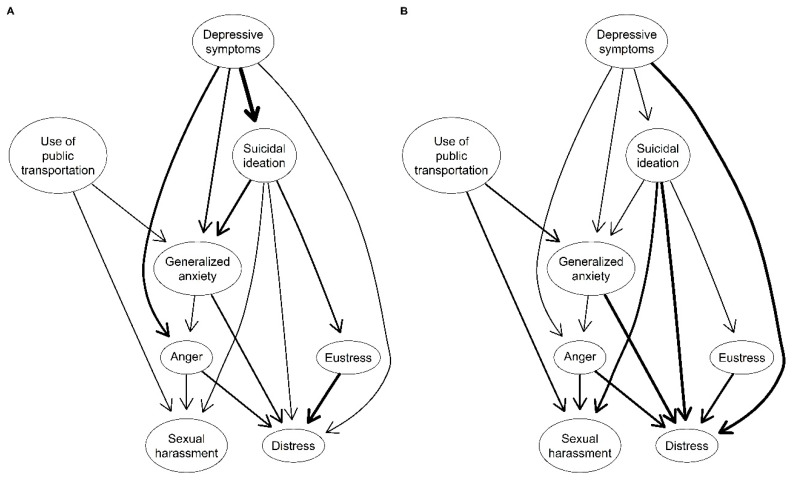
DAG of public transportation use, sexual harassment, and mental health among adults from the general population. (**A**) represents the overall model fit, where thicker arrows indicate greater contribution to model adjustment. (**B**) shows the directional probability, where thicker arrows indicate a higher proportion of bootstrapped networks in which the arrow appeared in that direction.

Regarding model fit, Table 3 and Figure 4A show that the most relevant directed arrows contributing to the network structure were from depressive symptoms to suicidal ideation (BIC = −64.817), from eustress to distress (BIC = −49.846), from suicidal ideation to generalized anxiety (BIC = −38.392), and from depressive symptoms to anger (BIC = −32.974).

As for directional probability, Table 3 and Figure 4B identify the strongest directed arrow from suicidal ideation to distress (DP = 0.931), meaning that the arrow was present in that direction in 93.1% of the bootstrap networks, and only 6.9% in the opposite direction. Similarly, strong directed arrows were identified from depressive symptoms to distress (DP = 0.914) and from generalized anxiety to distress (DP = 0.900).

In terms of cascading structure, Figure 4B shows two parent nodes: depressive symptoms (in-degree = 0, out-degree = 4) and public transportation use (in-degree = 0, out-degree = 2). That is, adults with depressive symptoms are statistically more likely to experience anger, generalized anxiety, suicidal ideation, and distress. Meanwhile, adults who use public transportation are statistically more likely to experience generalized anxiety and sexual harassment. Finally, distress and sexual harassment were located at the bottom of the network structure. This suggests that distress results from conditional dependence on anger, generalized anxiety, suicidal ideation, and eustress. Similarly, sexual harassment results from conditional dependence on anger, suicidal ideation, and public transportation use.

## 4. Discussion

This study analyzed the relationships between public transportation use, sexual harassment, and mental health through both directed and undirected network models. To our knowledge, this is the first study to examine directional dependencies using a combined approach of Gaussian and directed acyclic graph models in a general adult population.

In particular, participants reported spending between 1 and 1.9 h per day on public transportation, and a significant proportion indicated having been victims of sexual harassment during their commutes, with most perpetrators being fellow passengers. Similar results have been reported in other Latin American cities with overloaded transit systems, such as Asunción, Bogotá, and Mexico City, where traffic congestion, peripheral urban layouts, and limited transportation efficiency may explain the extended travel times (Guzman & Bocarejo, 2017; Sabogal-Cardona et al., 2021; Torales et al., 2024). These findings also align with previous studies on sexual harassment in public transportation, particularly involving passengers as aggressors, since lack of surveillance, overcrowding, and traffic saturation create conditions in which perpetrators can act with impunity in public spaces (Boyer, 2021; Östergren et al., 2022; Sabogal-Cardona et al., 2021). Lastly, the predominantly poor or fair perceptions of hygiene, comfort, and driver behavior, along with a particularly negative perception of safety, reveal a generally low level of satisfaction with public transportation services. These findings are consistent with the literature linking user dissatisfaction to poor maintenance, service quality, and public safety concerns, underscoring the need for comprehensive reform in the management and regulation of public transport (Conceição et al., 2023; Sabogal-Cardona et al., 2021; Torales et al., 2024).

In the undirected network structure, generalized anxiety, depressive symptoms, and anger emerged as central symptoms. This suggests these constructs are fundamental to emotional distress and may be explained by repeated exposure to social stressors such as long commutes, insecurity, and sexual harassment in public transport (Iamtrakul et al., 2024; Lan et al., 2020; Ye et al., 2023). Observed network relationships indicated that public transportation use was directly linked to negative emotional experiences such as anger and sexual harassment, reinforcing the notion that urban transport conditions affect not only time and safety but also users’ mental health. Specifically, anger may be driven by factors like overload, crowding, delays, and perceived mistreatment by drivers or other passengers—conditions often reported in studies on transportation-related stress (Conceição et al., 2023; Ye et al., 2023). Sexual harassment findings are also consistent with studies documenting high rates of gender-based violence in public transit, which result in significant and lasting emotional impacts (Anwar et al., 2022; Nath et al., 2022; Nguyen et al., 2025; Östergren et al., 2022).

In the directed network structure, public transportation use and depressive symptoms emerged as parent nodes, indicating that they act as triggers or initiating factors for other emotional conditions. Specifically, public transportation use was conditionally associated with increased likelihood of generalized anxiety and sexual harassment. This finding is supported by studies linking exposure to crowded public spaces with stress, insecurity, and violence—especially in urban contexts where harassment in transit systems is a recognized issue (Anwar et al., 2022; Nath et al., 2022; Östergren et al., 2022). In this context, generalized anxiety may serve as an anticipatory response to perceived threats or past experiences of harassment or violence in public spaces (Lan et al., 2020). Meanwhile, depressive symptoms were statistically associated with a higher likelihood of experiencing anger, generalized anxiety, suicidal ideation, and distress. This pattern is consistent with the previous literature that identifies depression not only as a standalone condition but also as a significant predictor of other forms of emotional distress (Conceição et al., 2023; Iamtrakul et al., 2024; Ye et al., 2023). Both distress and sexual harassment were positioned at the bottom of the network, suggesting that these variables function as outcomes of multiple preceding conditions. This highlights the importance of mental health in public urban mobility environments for the general adult population (Boyer, 2021; Gekoski et al., 2017).

The implications of this study extend to both mental health and urban planning and transportation policy. The findings underscore the need to consider contextual and structural factors in mental health prevention strategies. In urban environments, mobility should not be understood solely as an infrastructure issue but as a social determinant that directly impacts emotional well-being (Conceição et al., 2023; Guzman & Bocarejo, 2017). Identifying conditional relationships between public transportation use and experiences such as sexual harassment, generalized anxiety, and anger calls for the development of psychosocial interventions to reduce exposure to risk. These may include strengthening safety and mental health protocols, implementing campaigns to prevent sexual harassment, ensuring visible presence of trained personnel in transport systems and stations, and improving infrastructure to reduce overcrowding. The link between environmental urban conditions and emotional distress reinforces the need to incorporate mental health considerations into mobility plans—where quality indicators for transportation should include not only operational efficiency but also user-perceived safety, comfort, hygiene, and satisfaction.

Beyond general recommendations, the network results provide theoretically grounded and precise intervention points. The identification of sexual harassment as a conditional factor strongly linked to symptoms such as anxiety and anger suggests that reducing exposure to harassment may have cascading effects on broader emotional well-being. Policies aimed at prevention campaigns, improved reporting systems, and the visible presence of trained staff in transport systems are therefore not only protective measures but also mechanisms that can indirectly reduce central symptoms in the network. Similarly, the finding that anxiety and depression emerged as central nodes highlights the importance of implementing accessible mental health support integrated within mobility systems, for instance, through crisis hotlines, psychoeducational campaigns, or referral pathways from transport authorities to mental health services. Addressing these central symptoms may disrupt pathways of influence that connect contextual stressors to wider psychological distress. In parallel, the prominence of anger in conditional associations suggests that interventions to reduce overcrowding, improve infrastructure, and enhance user comfort may directly mitigate emotionally charged responses that feed into negative cycles of distress.

At the same time, it is important to acknowledge the cultural and socioeconomic specificity of this study. The data were drawn from Peru, a Latin-American country where urban transport systems are often marked by high levels of informality, gender-based inequalities, and limited institutional capacity to address harassment and safety concerns. These contextual factors may amplify the impact of public transport experiences on mental health and shape the ways in which individuals perceive and cope with insecurity. While the mechanisms identified in this study—such as the central role of harassment in deteriorating emotional well-being—are relevant across contexts, the intensity and expression of these processes may vary depending on local cultural norms, socioeconomic conditions, and institutional frameworks. Therefore, caution is needed when generalizing the findings to other settings, and future research should test the robustness of these associations in diverse cultural and geographic contexts.

The strength of this study lies in its use of a combined approach involving directed and undirected network models, which are well suited for representing dynamic and complex relationships (Burger et al., 2023). However, several limitations should be noted. First, the cross-sectional design means that data were collected at a single time point, limiting causal inference. While the DAG provides an initial view of the intensity and direction of variable connections, caution must be exercised when interpreting potential causal relationships. Future studies should consider longitudinal designs to observe how these variables evolve over time. Second, the study employed non-probabilistic sampling, limiting the generalizability of results to the broader population. Probability-based sampling methods are recommended for achieving more representative samples. Third, the sample was not demographically balanced, with overrepresentation of women and online data collection. Future studies should aim for greater demographic homogeneity, including gender balance and the use of face-to-face or hybrid data collection methods to reduce potential bias. Fourth, the variables related to public transportation use, sexual harassment, and mental health were assessed using self-report measures, which rely on subjective perceptions and may be affected by social desirability bias. Complementing self-reports with objective measures is recommended to strengthen the validity of findings. Finally, some of the directional connections in the model were weak or bidirectional, suggesting ambiguous relationships. Future studies should employ temporal network models to validate these interactions (Briganti et al., 2023).

## 5. Conclusions

The study’s findings suggest that implementing preventive and psychosocial intervention strategies in the context of public transportation could significantly reduce the occurrence of sexual harassment and the emergence of mental health problems in the general adult population. These actions are essential for promoting safer, more inclusive, and healthier public environments.

## Figures and Tables

**Table 1:** Satisfaction with public transportation services among adults from the general population.

Satisfaction Level	General	Hygiene	Comfort	Safety	Driver Behavior
Poor	154 (30.4%)	223 (44.0%)	198 (39.1%)	265 (52.3%)	117 (23.1%)
Fair	287 (56.6%)	225 (44.4%)	260 (51.3%)	200 (39.4%)	296 (58.4%)
Acceptable	60 (11.8%)	56 (11.0%)	45 (8.9%)	37 (7.3%)	78 (15.4%)
Good	6 (1.2%)	3 (0.6%)	4 (0.8%)	5 (1.0%)	16 (3.2%)
Excellent	0 (0%)	0 (0%)	0 (0%)	0 (0%)	0 (0%)

**Table 2:** Descriptive statistics and local network properties.

Nodes	Mean (*M*)	SD (*SD*)	Expected Influence (*EI*)	Predictability (*P*)
Use of public transportation	32.15	7.04	0.14	19.7%
Anger	9.21	3.69	0.91	39.8%
Eustress	12.84	3.03	0.22	26.7%
Distress	9.46	2.79	0.18	39.4%
Suicidal ideation	7.32	4.08	0.62	39.6%
Generalized anxiety	1.25	1.55	1.05	41.7%
Depressive symptoms	1.20	1.46	0.93	39.6%
Sexual harassment	0.78	0.42	−0.48	79.3%

**Note.** *M* = mean, *SD* = standard deviation; *EI* = Expected Influence, *P* = predictability.

**Table 3:** BIC values and directional probabilities of arrows in the DAG.

From	To	BIC	DP
Use of public transportation	Generalized anxiety	−6.206	0.703
Use of public transportation	Sexual harassment	−2.790	0.749
Anger	Distress	−14.034	0.812
Anger	Sexual harassment	−2.952	0.693
Eustress	Distress	−49.846	0.812
Suicidal ideation	Eustress	−19.314	0.627
Suicidal ideation	Distress	−1.563	0.931
Suicidal ideation	Generalized anxiety	−38.392	0.589
Suicidal ideation	Sexual harassment	−4.876	0.800
Generalized anxiety	Anger	−7.734	0.536
Generalized anxiety	Distress	−15.289	0.900
Depressive symptoms	Anger	−32.974	0.648
Depressive symptoms	Distress	−0.676	0.914
Depressive symptoms	Suicidal ideation	−64.817	0.534
Depressive symptoms	Generalized anxiety	−17.216	0.599

**Note.** BIC = Bayesian Information Criterion; DP = directional probability. Negative BIC values indicate that the model fit improves with the inclusion of the respective arrow. Directional probability values reflect the proportion of 10,000 bootstrapped networks in which the arrow appeared in that direction.

## Data Availability

The data presented in this study are available upon request from the corresponding author due to privacy issues.

## Abbreviations

The following abbreviations are used in this manuscript:

EBIC	Extended Bayesian Information Criterion
EI	Expected Influence
APL	Average Path Length
SC	Stability Coefficient
DAG	Directed Acyclic Graph
HC	Hill-Climbing
BIC	Bayesian Information Criterion
